# tBHQ Induces a Hormetic Response That Protects L6 Myoblasts against the Toxic Effect of Palmitate

**DOI:** 10.1155/2020/3123268

**Published:** 2020-05-16

**Authors:** Pedro Posadas-Rodríguez, Natalia Esmeralda Posadas-Rodríguez, Viridiana Yazmín González-Puertos, Rafael Toledo-Pérez, José Luis Ventura-Gallegos, Alejandro Zentella, Luis Enrique Gómez-Quiroz, Mina Königsberg, Armando Luna-López

**Affiliations:** ^1^Departamento de Ciencias de la Salud, Universidad Autónoma Metropolitana-Iztapalapa, Ciudad de México, Mexico; ^2^Posgrado en Biología Experimental, Universidad Autónoma Metropolitana-Iztapalapa, Ciudad de México, Mexico; ^3^Departamento Medicina Genómica y Toxicología Ambiental, IIB, UNAM, México, DF, Mexico; ^4^Departamento Bioquímica, Instituto Nacional de Ciencias Médicas y Nutrición “Salvador Zubirán”, México, DF, Mexico; ^5^Departamento de Investigación Básica, Instituto Nacional de Geriatría, SSA, Ciudad de México, Mexico

## Abstract

Nutritional status, in particular overweight and obesity, as well as sedentarism and high-fat diet consumption, are important risk factors to develop chronic diseases, which have a higher impact on the elderly's health. Therefore, these nutritional problems have become a concern to human healthspan and longevity. The fatty acids obtained thru the diet or due to fatty acid synthesis during obesity accumulate within the body generating toxicity and cell death. Fat is not only stored in adipose tissue, but it can also be stored in skeletal muscle. Palmitic acid (PA) has been reported as one of the most important saturated free fatty acids; it is associated to chronic oxidative stress and increased mitochondrial ROS production causing cell death by apoptosis. In skeletal muscle, palmitate has been associated with various pathophysiological consequences, which lead to muscle deterioration during aging and obesity. Since molecules that modify redox state have been proven to prevent cellular damage by inducing a hormetic response, the aim of this study was to evaluate if tert-butylhydroquinone (tBHQ) could activate an antioxidant hormetic response that would be able to protect L6 myoblasts from palmitate toxic effect. Our results provide evidence that tBHQ is able to protect L6 myoblasts against the toxicity induced by sodium palmitate due to a synergistic activation of different signaling pathways such as Nrf2 and NF-*κ*B.

## 1. Introduction

Modern lifestyle and particularly the increased consumption of high-fat diets have become a topic of concern when it comes to healthspan. It is known that nutritional status, in particular overweight and obesity, are important risk factors to develop chronic diseases such as type 2 diabetes, cardiovascular disease, cancer, and dementias among others [1–3], which regrettably have a higher impact on older people's health [4, 5]. It has been shown that obese people live an important part of their life in suboptimal health conditions and their life expectancy is reduced [6, 7]. Therefore, the obesity pandemic has emerged as an important challenge to human healthspan and longevity [5, 8].

Dietary fatty acids or fatty acids from de novo synthesis during obesity accumulate within the body. Fat is not only stored in adipose tissue, but it could also be stored in skeletal muscle tissue [9]. Some reports have shown that fatty acids have an important role in oxidative damage and skeletal muscle deterioration [10, 11]. After muscle damage and degeneration, a new regenerative cycle restores the muscle fibers at the expense of resident myogenic cells. This regeneration is mainly sustained by the myoblasts [12]; their fusion is a critical process that contributes to muscle growth during development and myofiber regeneration after injury [13]; and nevertheless, during obesity, this event is compromised due to inflammation and oxidative stress [14]. Moreover, hyperglycemia and high levels of circulating free fatty acids (FFA) have been related to chronic oxidative stress and increased mitochondrial ROS production [15–17].

Palmitic acid (PA) is the most abundant saturated FFA found in processed food and is one of the most abundant FFA in human and rodent plasma, representing 22–25% of the total FFA [18, 19]. In the skeletal muscle, PA, or its deprotonated form palmitate, has been reported to increase apoptosis through different mechanisms, including endoplasmic reticulum (ER) stress, mitochondrial dysfunction and oxidative damage, JNK activation, and lysosomal membrane permeabilization [20–22]. It has been recently demonstrated that myoblasts are more sensitive to palmitate deleterious effects than myotubes and that some antioxidants like MitoTEMPOL prevented palmitate-induced mtDNA damage but could not prevent cell death [23]. Therefore, since palmitate has been associated with various pathophysiological consequences in skeletal muscle, here we studied whether inducing an antioxidant hormetic response could protect myoblasts from sodium palmitate-induced cellular damage.

“Hormesis is a process in which exposure to a low dose of a chemical agent or environmental factor that is damaging at higher doses induces an adaptive beneficial effect on the cell or organism” [24, 25]. This process gained special attention in 1986 when Murry et al. showed that a small ischemic stress reduced heart tissue damage by about 75% in dogs subsequently subjected to prolonged ischemia [26–28].

Our group has previously used the compound tert-butylhydroquinone (tBHQ) as a hormetic inducer [29, 30]. tBHQ is a synthetic phenol widely used as a food preservative and antioxidant. Besides, tBHQ has also been shown to act as a prooxidant and generate ROS, in order to activate the redox response in a hormetic manner activating the survival response [31–33]. Numerous evidence has demonstrated that tBHQ is effective in protecting cells against dysfunction provoked by oxidative stress inducers, such as alcohol, dopamine, hydrogen peroxide, 3NP (3-nitropropionic acid), and MPTP (1-methyl-4-phenyl-1,2,3,6-tetrahydropyridine), in various cell types and animal models [29, 30, 34–36]. As mentioned above, a hormetic inducer has the ability to turn on several adaptive responses (survival, proliferation, cell differentiation, etc.) in order to cope with stress. It has been well established that tBHQ activates the nuclear factor (erythroid-derived 2)-like 2 (Nrf2), via the inhibition of the Kelch-like ECH-associated protein 1- (Keap1-) mediated ubiquitination [34, 36, 37]. Hence, the aim of this work was to evaluate tBHQ effect and mechanism of action as a hormetic inducer to protect the myoblast cell line against palmitate toxicity.

## 2. Materials and Methods

### 2.1. Chemicals

All chemicals and reagents were purchased from Sigma Chemical Co. (St. Louis, MO). The reagents obtained from other sources are detailed throughout the text.

### 2.2. L6 Cell Culture

L6 cells are myoblasts originally obtained from rat (*Rattus norvegicus*) skeletal muscle and were purchased from ATCC (CRL1458). For this study, L6 cells were routinely cultivated in Dulbecco's modified Eagle's medium (DMEM) supplemented with 10% fetal bovine serum (FBS, Corning Inc., Corning, NY) at 37°C and 5% CO_2_. Since L6 are normal myogenic cells, they tend to fuse in culture to form multinucleated myotubes and striated fibers; therefore, L6 cells were always reseeded by 60% confluence to avoid myotubule formation.

### 2.3. Cell Viability

To determine L6 cell viability under the different conditions, trypan blue assay was performed as described before [38]. Cells were counted using the TC20 automated cell counter (Bio-Rad).

### 2.4. Fatty Acid Preparation

Palmitate is the most prominent saturated fatty acid utilized in the body. However, the utilization of palmitate in cell-based assays is challenging due to its low solubility in aqueous solutions. Since palmitate conjugation to bovine serum albumin (BSA) creates an aqueous-soluble reagent that can be absorbed and utilized by cells, it has been usually used as a transporter and stabilizing agent for fatty acids for that kind of experiments [39]. For the preparation of sodium palmitate-BSA solution, the next conjugation was made, 5 mM sodium palmitate/0.85 mM BSA solution (6 : 1 molar ratio palmitate : BSA). Palmitate was conjugated to BSA when both components were solubilized. Briefly, sodium palmitate was solubilized in 150 mM sodium chloride by heating up to 65°C in a water bath. BSA was dissolved in phosphate-buffered saline (PBS) and warmed up to 37°C, with continuous stirring, and filtered with a 150 mL filter unit in a vacuum. Solubilized sodium palmitate was added to BSA at 37°C with continuous stirring. The pH was adjusted to 7.4 with 1 N NaOH. The conjugated sodium palmitate-BSA (which will be further referred as palmitate) was aliquoted and stored at −20°C. The working solutions used in the experiments were 0.25 mM, 0.75 mM, and 1 mM.

### 2.5. Neutral Lipid Determination

After 24 h of incubation with palmitate solution, L6 cells were washed two times with phosphate-buffered saline (PBS), fixed in paraformaldehyde 4% for 10 min, and stained with 0.2% freshly prepared Oil Red O (ORO) solution for 1 h. After rinsing with PBS, cells were counterstained with hematoxylin. Cell stained images were obtained under a microscope (AxioVert A1, Carl Zeiss). After obtaining representative images, ORO was extracted using 1 mL of isopropyl alcohol for 10 min and optical density measurement was done at wavelength 510 nm and normalized with the cellular number of each treatment.

### 2.6. Immunofluorescence Experiments

Myoblasts were washed with PBS and fixed with 4% paraformaldehyde for 15 min. Immediately thereafter, cells were incubated in universal blocking reagent (BioGenex) for 15 min at room temperature. Cells were washed and incubated for 1 h with the primary antibodies: myogenin and Myf-5 (SC127132 and SC518039, Santa Cruz Biotechnology, Santa Cruz, CA, USA) and pNrf2 and p65 (76026 and 16502, Abcam, Cambridge, MA). Cells were washed 3 times with PBS-Tween 0.2% and were incubated with the corresponding secondary antibody. After 2 more washes, cells were further incubated with DAPI (10 *μ*g/mL) for 10 min to stain the DNA and mark the nucleus. Single-plane images were obtained with a confocal microscope LSM-META-Zeiss Axioplan 2 imaging at 30x and analyzed using the ZEN 2010 program version 6.0 (Carl Zeiss).

### 2.7. Western Blot Analysis

Treated and untreated L6 myoblasts were trypsinized and resuspended in lysis buffer M-PER (Pierce Chemical, Rockford, IL, USA) supplemented with protease inhibitors (complete; Roche Applied Science, Indianapolis, IN, USA), 1 mM phenylmethylsulfonyl fluoride (PMSF), and 0.1 mM dithiothreitol (DTT). Cell homogenates were incubated at 4°C for 5–10 min and centrifuged at 14,000 × *g*, 4°C for 20 min. Protein concentration was determined in the supernatants using a commercial Bradford reagent (Bio-Rad, Hercules, CA, USA) [40]. To prepare the samples for the SDS-PAGE, the Laemmli buffer (Bio-Rad, Hercules, CA, USA) was used along with the reducing agent 2-mercaptoethanol (Bio-Rad, Hercules, CA, USA). The samples were mixed and then heated at 90–100°C for 5 min and subsequently left on ice for 5 min. Then, 30 *μ*g of the protein sample was loaded on the gel. Cell lysates were separated on 12% sodium dodecyl sulfate-polyacrylamide gel electrophoresis (SDS-PAGE) and transferred to polyvinylidene difluoride membranes (Invitrogen, Waltham, MA, USA), using a semidry blotting process (Trans-Blot Turbo System; Bio-Rad, Hercules, CA, USA). The membranes were then blocked with 10% BSA in PBS for 1 h followed by excess removal with TBS-Tween rinses, then probed with primary antibodies specific to pNrf2 (1 : 500, Ab76026), p65 (1 : 500, Ab16502) (Abcam, Cambridge, MA), lamin A (1 : 1000, SC518013), heme oxygenase-1 (HO-1) (1 : 1000, SC136960), glutathione sulfhydryl transferase (GST) (1 : 500, SC1389), *γ*-glutamylcysteine synthetase (*γ*-GCS) (1 : 500, SC55586), glutathione reductase (GR) (1 : 500, SC56851), superoxide dismutase-1 (SOD-1, 1 : 500, SC17767), catalase (CAT) (1 : 500, SC271803), Bcl-2-associated X protein (Bax) (1 : 500, SC7480), heat shock protein-70 (HSP70, 1 : 1000, SC32239), and actin (1 : 1000, SC47778) (Santa Cruz Biotechnology, Santa Cruz, CA, USA). The antibodies were incubated overnight at 2-8°C; the membranes were washed 3 times with TBS-Tween and incubated for 2 h with the secondary antibody anti-rabbit (SC2357), anti-goat (SC2768), or anti-mouse (SC358914) at a dilution 1 : 1000; antibodies were purchased from Santa Cruz Biotechnology (Santa Cruz, CA). The membranes were then washed 3 times with TBS-Tween horseradish peroxidase-conjugated secondary antibodies (Pierce, Rockford, IL, USA) for 1 h. After the 3 washes, the blots were revealed using a commercial chemiluminescent reagent (SuperSignal Pierce, Rockford, IL, USA). The development was carried out in the Western blot and chemiluminescence imaging system (FUSION FX Vilber Lourmat, Vilber smart imaging).

### 2.8. Redox State (GSH/GSSG Ratio)

The content of GSH and GSSG was determined as described by Hernández-Álvarez et al. (2019) with some modifications. Treated cells were trypsinized and homogenized in hydrochloric acid/BPDS (HCl 10%/BPDS 1 mM) and centrifuged at 5000 × *g* for 5 min a 4°C. The supernatant was recovered, and 100 *μ*L of each sample was injected into the HPLC system. A 1525 Waters binary pump coupled to an UV/Vis 2489 (210 nm) was used. The stationary phase used was a ZORBAX Eclipse XDB-C18, 4.6 × 250 mm, 5 *μ*m column with acetonitrile 1% and potassium phosphate monobasic buffer 99% (20 mM KH_2_PO_4_; pH 2.7). An isocratic flow of 1 mL/min was used. The samples were analyzed by ultraviolet detection at 210 nm. The area under the curve was determined by using GSH and GSSG standards, starting with different concentrations (10, 25, 50, 100, 200, and 400 *μ*M).

### 2.9. Statistical Analysis

All experiments represent at least 3 independent experiments per group. Data are reported as mean values ± standard error, and results were analyzed by means of a parametric 1-way analysis of variance followed by a Tukey–Kramer test, using the GraphPad Prism 6.01. The level of confidence *p* < 0.05 was considered statistically significant.

## 3. Results

### 3.1. Cell Culture Characterization

**Figure 1:**
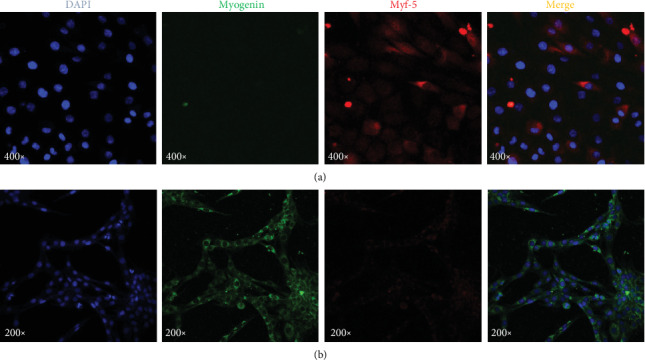
L6 cell line characterization. The figure shows representative images of three independent cultures in triplicate. The cells were treated as described in Materials and Methods. Myoblasts are positive to Myf-5 (red) and negative for myogenin (green), while myotubes are stained in the opposite way. L6 were cultured in basal conditions to maintain myoblast phenotype. L6 were cultured with horse serum to induce their differentiation into myotubes.

The L6 myoblast cell line is prone to terminally differentiate into skeletal muscle myotubes by altering the culture environment (low serum conditions or high confluence levels). The process of myoblast fusion is critical for skeletal muscle myotube development. During the process of myoblast differentiation, the myogenic cells exit the cell cycle and mitotic activity ceases [41, 42]. For this reason, the formation of myotubes in this study was avoided, whereby the experiments were always performed at 60% confluence. To verify the culture conditions, the myogenic regulatory factors (MRFs) Myf-5 and myogenin were determined as specific differentiation markers, because Myf-5 is involved in the early stages of differentiation, while myogenin regulates the later stages of myogenic differentiation [43]. The results shown in Figure 1(a) confirm that the conditions under which the L6 cells were cultured preserved the myoblasts stage and did not generate myotubes. On the other hand, to evaluate if the cells preserved their capacity to differentiate, they were subjected to conditions that encourage their differentiation. As shown in Figure 1(b), L6 cells grown in the medium with horse serum developed myotubes; as expected, the cells expressed myogenin and not Myf-5.

### 3.2. L6 Myoblast Susceptibility to tBHQ and Changes in Redox State

**Figure 2:**
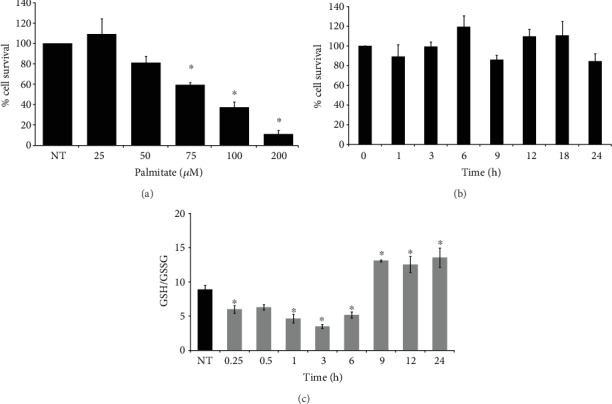
L6 viability and redox state. (a) L6 myoblasts were treated with different concentrations of tBHQ (20, 50, 75, 100, and 200 *μ*M) during 24 h, and the percentage of cellular survival at those concentrations was determined as described in Materials and Methods. (b) Since 50 *μ*M tBHQ was chosen for further experiments, a time course-response curve using that concentration was performed at 1, 3, 6, 9, 12, 18, and 24 h. Cell viability was determined as described in Materials and Methods. (c) GSH/GSSG ratio was evaluated as described in Materials and Methods at different time points during 24 h in L6 cells treated with tBHQ 50 *μ*M. Each bar represents the mean ± S.E. of 9 determinations performed in three independent experiments. Statistical significance with respect to nontreated cells ^∗^*p* < 0.05.

A dose-response curve for tBHQ was performed to find the possible hormetic concentration to be used. L6 myoblasts were treated with progressive tBHQ concentrations (20, 50, 75, 100, and 200 *μ*M) for 24 h.  Figure 2(a) shows the percentage of cells that survived after tBHQ treatment. No differences were observed at 20 and 50 *μ*M tBHQ treatments compared to the control, with the last one being the maximum concentration without generating a significant decrease in cell death, and it was therefore chosen for further experiments. Conversely, with 75 *μ*M tBHQ treatment, the percentage of living cells was 59.5% with respect to the control, while during 100 *μ*M treatment, only 37.5% survived and the 200 *μ*M concentration practically killed all the cells. Then, a time course-response curve was performed with 50 *μ*M tBHQ and cellular survival was measured at 1, 3, 6, 9, 12, 18, and 24 h.  Figure 2(b) shows that no significant differences were found during that time.

It is known that tBHQ is associated with redox changes [44] because its self-oxidation generates tert-butylbenzoquinone (TBQ) which is associated with reactive oxygen species (ROS) generation. So the next step was to elucidate the redox state in myoblasts after tBHQ treatment. Redox state was measured as the GSH/GSSG ratio (Figure 2(c)). L6 myoblast cells were treated with tBHQ 50 *μ*M during 0.25, 0.5, 1, 3, 6, 9, 12, and 24 h. During the first hours (0.25-6 h), the redox state was more oxidized compared to the control cells, while at 9, 12, and 24 h, there was a shift towards a more reduced state, suggesting that at those times the system might be protected against oxidative damage.

### 3.3. Effect of Palmitate on L6 Myoblast Viability

**Figure 3:**
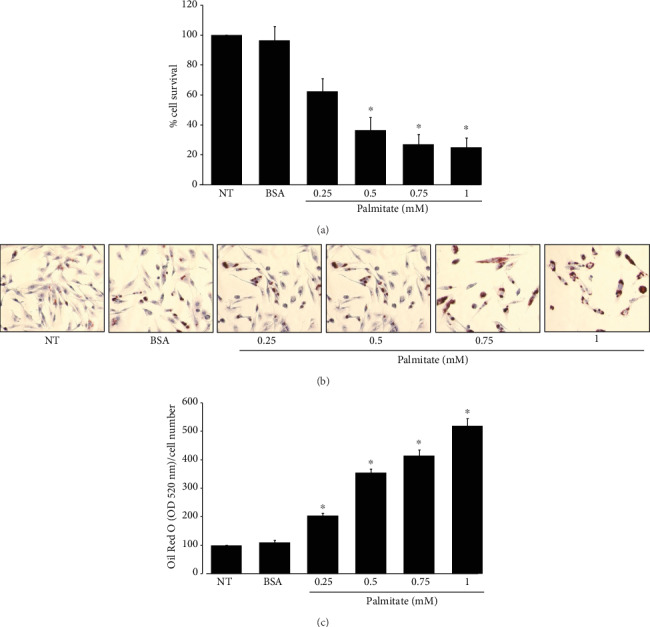
Palmitate induced cell death. L6 myoblasts were treated with different concentrations of palmitate for 24 h. (a) Cell viability. (b) ORO staining of lipid droplets was evaluated. (c) Spectrophotometric quantification of ORO stain to determine intracellular lipid load. Each bar represents the mean ± S.E. of 9 determinations performed in three independent experiments. Statistical significance with respect to untreated cells ^∗^*p* < 0.05.

Cells were exposed to palmitate-BSA conjugate at different concentrations, and L6 cell survival was determined after 24 h (Figure 3(a)). Treatment with palmitate 0.25 mM did not show significant differences with respect to the untreated cells. Palmitate 0.5 mM resulted in 64% of cell death at 24 h compared to the control, while for 0.75 and 1 mM, cellular demise was 73 and 75%, respectively. Therefore, the concentration of palmitate 0.5 mM was chosen for further experiments.

To verify the palmitate internalization into the cells, the cultures were incubated with palmitate-BSA conjugate at different concentrations for 24 h and subsequently treated with ORO as described above.  Figure 3(b) shows that palmitate internalization in the cells (red staining) augmented proportionally to increased concentration. Interestingly, at the higher palmitate concentrations (0.75 and 1 mM), a decrease in the number of cells is observed, which correlates with cell death (Figure 3(a)).

To quantitatively determine the amount of fatty acids incorporated during each treatment, the ORO was extracted and normalized with respect to the number of cells (Figure 3(c)). The cells treated with 0.25 mM palmitate contained twice the lipid load of the control cells; 0.5 mM palmitate-treated cells showed 3.5 times more than controls, while for 0.75 mM palmitate cells, the increment was 4.15-fold and finally for 1 mM, the increase was 5.1-fold with regard to the untreated cells.

### 3.4. tBHQ Protects against Palmitic Acid-Induced Cell Death in L6 Myoblasts

**Figure 4:**
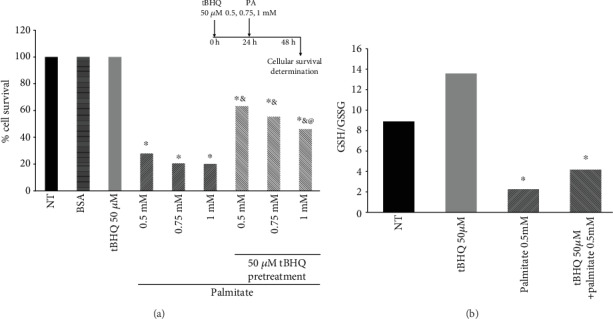
Cell survival and redox state after palmitate insult following the hormetic treatment. (a) Cell survival was determined after 24 h of palmitate insult (0.5, 0.75, or 1 mM) following 24 h of tBHQ 50 *μ*M pretreatment or no treatment. The insert in this figure represents the experimental design used. Each bar represents the mean ± S.E. of 9 determinations performed in three independent experiments. Statistical significance with respect to controls (NT, BSA, and tBHQ) ^∗^*p* < 0.05; ^&^different from palmitate-treated cells without hormetic treatment; ^@^different from the 0.5 mM palmitate-treated hormetic cells. (b) GSH/GSSG ratio was measured as described in Materials and Methods after 24 h of 0.5 mM PA insult following 24 h of tBHQ 50 *μ*M hormetic pretreatment. Each bar represents the mean ± S.E. of 9 determinations performed in three independent experiments. Statistical significance with respect to untreated cells ^∗^*p* < 0.05.

To evaluate if the tBHQ hormetic pretreatment protects L6 cells against palmitate harmful effects, we evaluated cellular survival after 24 h of palmitate exposure to different concentrations. The insert in Figure 4(a) shows the experimental design used. Cells treated only with BSA (palmitate vehicle) or tBHQ were used as complementary controls. As shown in the figure, tBHQ 50 *μ*M pretreatment was able to prevent cell death when compared to L6 subjected to palmitate insult but not to the hormetic treatment. L6 treated with palmitate showed approximately 72% of cell death compared to those treated hormetically, where death was 35 to 55% depending on the palmitate concentration used, since there was a statistically significant difference between the cells treated with 0.5 mM palmitate (35% cell death) and the ones treated with 1 mM palmitate (55%). Still, both of them were significantly higher than the cells that did not receive the hormetic treatment.

Besides the viability experiments, we were also interested in assessing the redox state in the hormetic model; therefore, the GSH/GSSG ratio was determined. This experiment was performed only in L6 myoblasts subjected to palmitate 0.5 mM, since those cells had the highest percentage of protection.  Figure 4(b) shows a significant increase in the GSH/GSSG ratio (50%) with respect to the cells subjected to palmitate without the hormetic treatment. Hence, the improvement in redox state besides the viability protection supports the idea that tBHQ induces a hormetic protection against palmitate in L6 cells.

### 3.5. Nrf2 Activation due to tBHQ Treatment

**Figure 5:**
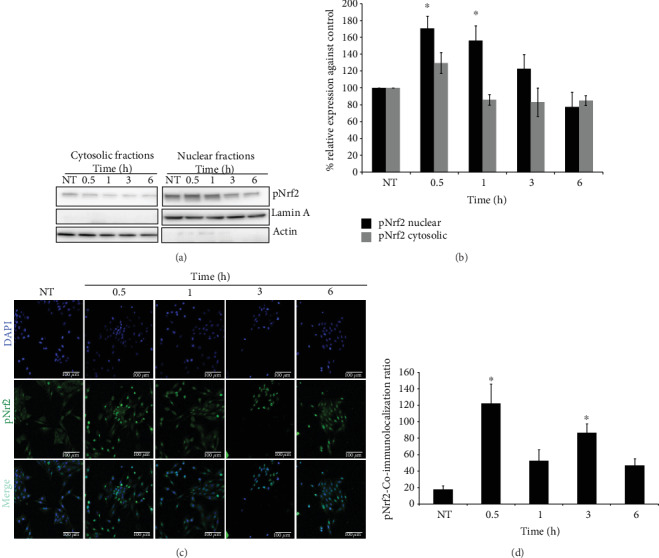
Nrf2 activation and nuclear translocation. (a) Representative Western blot images of pNrf2 in nuclear and cytosolic fractions in the L6 cell line after 0.5, 1, 3, and 6 h of tBHQ 50 *μ*M treatment. (b) Densitometric analysis of nuclear and cytosolic fractions of pNrf2 immunoblots. (c) Representative immunofluorescences of the L6 cell line after 0.5, 1, 3, and 6 h of tBHQ 50 *μ*M treatment. The nucleus is marked with DAPI (blue) and pNrf2 (green). (d) pNrf2-nuclear coimmunolocalization ratio. Data represents the mean ± S.E. of 9 determinations performed in three independent experiments. Statistical significance with respect to untreated cells ^∗^*p* < 0.05.

To determine the early events that tBHQ triggers in order to induce protection, we determined Nrf2 activation and nuclear translocation at early time points (0.5 and 1 h) after tBHQ 50 *μ*M treatment (Figure 5). Our data showed that no significant increase in pNrf2 was found in the cytosolic fraction with respect to the untreated cells. However, a significant increase of 1.7- and 1.5-fold with respect to the control was found in the nuclear fraction after 0.5 and 1 h of treatment, respectively (Figures 5(a) and 5(b)). To confirm these data, pNrf2 nuclear translocation was assessed by immunofluorescence in a timeline course from 30 min to 6 h during tBHQ treatment (0.5, 1, 3, and 6 h).  Figure 5(c) shows representative images that demonstrate pNrf2 location during tBHQ treatment. To evaluate nuclear location, the colocalization of pNrf2 with DAPI was performed using the Zen 2 lite software (Figure 5(d)). Interestingly, in contrast to the previous data, this experiment shows an increase in pNrf2 in the nucleus at 0.5 and 3 h of tBHQ treatment.

### 3.6. Modifications in Nrf2 Target Proteins Involved in Oxidant Defense and Redox Signaling

**Figure 6:**
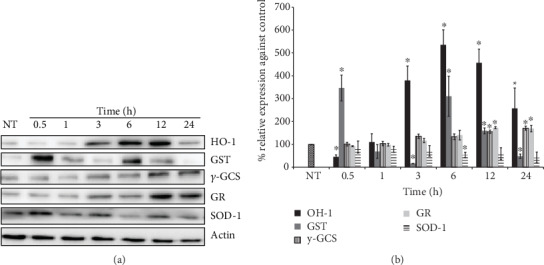
Nrf2 target proteins involved in oxidant defense and redox signaling. (a) Representative Western blot images of HO-1, GST, *γ*-GCS, GR, and SOD-1 after 0.5, 1, 3, 6, 12, and 24 h of 50 *μ*M tBHQ treatment. (b) Densitometric analysis of HO-1, GST, *γ*-GCS, GR, and SOD Western blots under the conditions mentioned above. The data were normalized against the basal protein levels (100%). Each bar represents the mean ± S.E. of 3 independent experiments. Statistical significance with respect to untreated cells ^∗^*p* < 0.05.

To confirm that Nrf2 activation and nuclear translocation were productive, the content of some proteins that are regulated by this transcription factor was evaluated after 0.5, 1, 3, 6, 12, and 24 h of tBHQ treatment (Figures 6(a) and 6(b)). The results were very interesting and different for the diverse proteins evaluated. In the case of heme oxygenase 1 (HO-1), it showed a gradual increase which reached its highest peak at 6 and 12 h and then started to decrease. However, at 24 h, HO-1 levels where still significantly higher than those in the untreated cells. For glutathione S-transferase (GST), there was a very rapid and significant increase at 0.5 h, which gradually declined until 3 h, when the protein showed its lower level, even lower than the control. Then, there was another increase at 6 h, followed by a decrease at 12 and 24 h. Gamma-glutamylcysteine synthetase (*γ*-GCS) and glutathione reductase (GR) showed the same behavior; their levels did not increase until 12 and 24, where they showed a slight but significant increase (1.0 times more than untreated cells). Interestingly, SOD-1 content did not significantly change during the treatments.

### 3.7. Nrf2 Is Not the Only Participant Responsible for tBHQ Protective Effect

**Figure 7:**
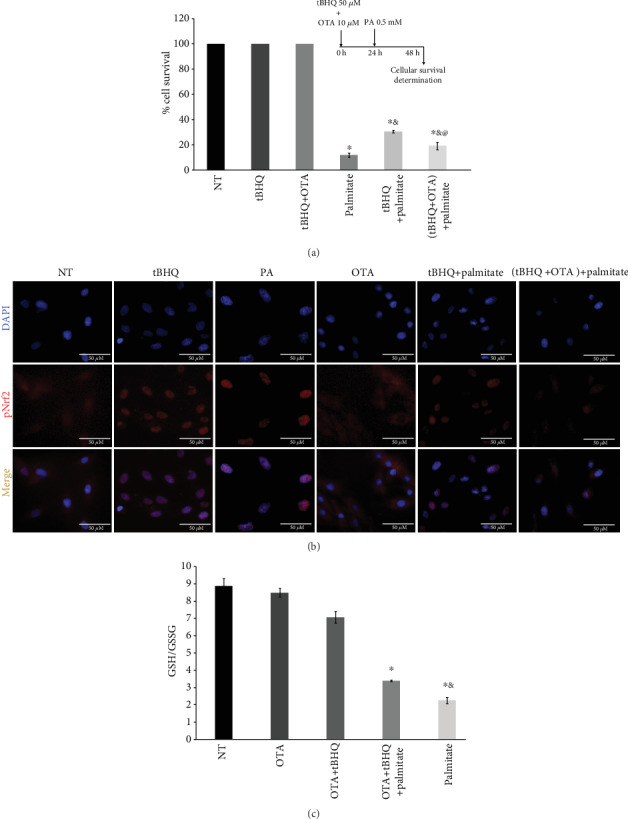
Inhibition of the hormetic model by Ochratoxin A (OTA). (a) Cell survival as described in Materials and Methods was evaluated after the insult with 0.5 mM palmitate to hormetic-treated L6 cells, with and without OTA 10 *μ*M, as shown in the insert. (b) Representative immunofluorescence images after OTA treatment in the hormetic model following the design described in (a) insert. pNrf2 (red) and DAPI (blue). (c) GSH/GSSG ratio was determined as described in Materials and Methods after the insult with 0.5 mM palmitate to the hormetic model following the design described in (a) insert. Each data represents the standard error of the mean of 9 determinations performed in 3 independent experiments. Statistical significance with respect to control cells ^∗^*p* < 0.05; ^&^different from palmitate-treated cells without hormetic treatment; ^@^different from tBHQ+palmitate-treated cells.

Since we found a differential induction in the proteins associated to antioxidant response regulated by Nrf2 associated to the tBHQ treatment, we decided to evaluate Nrf2 participation in the hormetic process. Therefore, we used Ochratoxin A (OTA), which is known to inhibit Nrf2 activation and gene transcription [45, 46]. First, we determined the OTA concentration to be used to inhibit Nrf2, looking for the minimum toxic concentration (Supplementary Fig. 1A). Moreover, to confirm Nrf2 nuclear translocation inhibition, immunolocalization assays were performed (Supplementary Fig. 1B). Since OTA 20, 50, and 100 *μ*M treatments killed more than 50% of the cells, the 10 *μ*M concentration was chosen for the Nrf2 inhibition experiments; so for those experiments, OTA 10 *μ*M was added to the hormetic pretreatment (tBHQ 50 *μ*M). L6 cells were pretreated for 24 h and then subjected to 0.5 mM palmitate for another 24 h, as illustrated in the insert of Figure 7(a). As seen in Figure 7(a), the results obtained in Figure 4(a) are repeated. In this case, it is observed that the treatment with palmitate induces 74% of cell death, while the hormetic treatment with tBHQ only kills 37% of the cell population. When OTA was added to the hormetic model, 63% of the cells were killed, suggesting that OTA abrogated the protective effect, but not completely since there was still a significant protection with the cells treated with palmitate. This implied that there could be other factors, different from Nrf2, participating in the hormetic response.

Figure 7(b) shows that when L6 cells were treated with OTA during the hormetic pretreatment, pNrf2 fails to translocate into the nucleus; the opposite is seen when the cells were treated only with tBHQ, where a pNrf2 is observed by colocalizing with the nucleus, confirming OTA inhibitory effect. In Figure 7(c), the redox state was evaluated through the GSH/GSSG ratio, and similar to the cell survival assay, we observe that the cells treated with OTA are more oxidized than the hormetic-treated cells but still different from the ones treated only with palmitate, suggesting again that Nrf2 is not the only participant during tBHQ protective effect.

**Figure 8:**
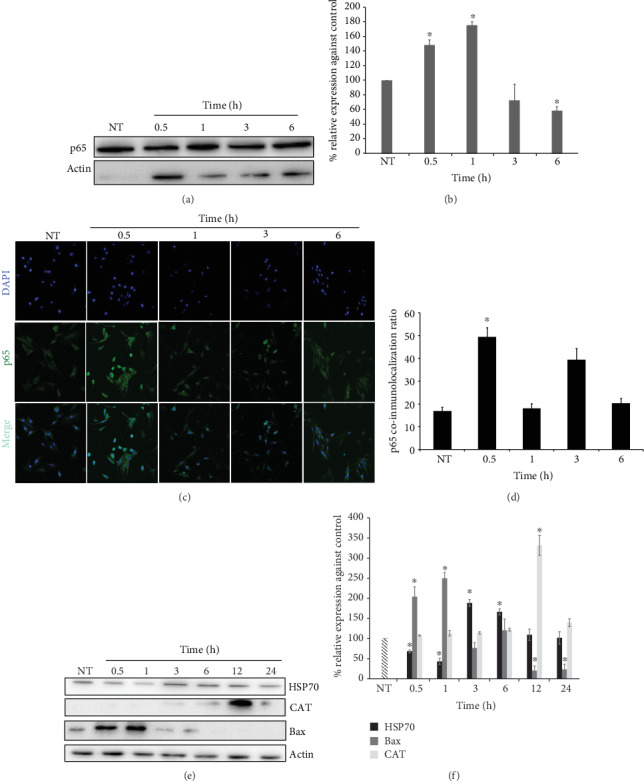
NF-*κ*B participation in the hormetic treatment. (a) Representative Western blot image of p65 after 0.5, 1, 3, and 6 h of 50 *μ*M tBHQ treatment. (b) Densitometric analysis of p65 Western blots under the conditions mentioned above. The data were normalized against the basal protein levels (100%). (c) Representative immunofluorescence images after 0.5, 1, 3, and 6 h of 50 *μ*M tBHQ treatment. p65 (green) and DAPI (blue). (d) p65 nuclear coimmunolocalization ratio 0.5, 1, 3, and 6 h of 50 *μ*M tBHQ treatment. (e) Representative Western blot images of HSP70, CAT, and Bax after 0.5, 1, 3, 6, 12, and 24 h of 50 *μ*M tBHQ treatment. (f) Densitometric analysis of HSP70, CAT, and Bax Western blots under the conditions mentioned above. The data were normalized against the basal protein levels (100%). Each bar represents the mean ± S.E. of 3 independent experiments. Statistical significance with respect to untreated cells ^∗^*p* < 0.05.

Another transcription factor that might be associated with the tBHQ hormetic effect is NF-*κ*B. This transcription factor is known for its sensitivity to cellular redox state; Sen and coworkers [47] demonstrated in L6 myoblasts that NF-*κ*B is activated by changes in the redox state associated to intracellular GSH/GSSG status. NF-*κ*B is a heterodimer composed of two related subunits, p65 and p50, which share a homologous region at the N-terminal end, necessary for DNA binding and dimerization [48]. Therefore, we evaluated the total p65 subunit in the lysates of L6 cells during the hormetic treatment at 0.5, 1, 3, and 6 h. Our results (Figure 8(a)) show a representative Western blot and its densitometric analysis (Figure 8(b)), where a significant increase of this NF-*κ*B subunit at 0.5 h and 1 h, with a later decrease at 6 h (*p* < 0.05), is observed. To confirm p65 participation in this protective process, its nuclear translocation was evaluated by immunofluorescence as illustrated in Figure 8(c). Moreover, p65 colocalization with the DNA marker is presented in Figure 8(b) that shows an increase in the signal corresponding to p65 colocalized in the nucleus at 0.5 h during tBHQ 50 *μ*M treatment, which correlates with the increase in p65 shown in the blot. To confirm the productivity of NF-*κ*B nuclear translocation, several proteins related to cell survival, such as HSP70, CAT, and Bax, were also determined by Western blot. Figures 8(e) and 8(f) show the increment in these proteins in response to the tBHQ 50 *μ*M treatment at different times (0.5, 1, 3, 6, 12, and 24 h). HSP70 decreased at 0.5 and 1 h, and it increased at 3 and 6 h. Bax displayed the opposite behavior; it significantly augmented at 3 and 6 h and then diminished at 12 and 24 h, while CAT only increased at 12 h, suggesting that the proteins' protective effect might be achieved at later times.

## 4. Discussion

Lipid infiltration into the muscle is a process associated with aging and obesity that induces muscular decline in quality and strength [49, 50]. Furthermore, adiposity excess and other obesity-mediated factors and pathways work synergistically to induce muscle atrophy. Palmitic acid (PA) is the most abundant saturated FFA in processed foods and is one of the most abundant FFA in human and rodent plasma [18, 51]. Since palmitate is known to induce lipotoxicity [11, 52, 53], it was interesting to determine if a hormetic model could decrease cell death due to this FFA.

The L6 myoblast cell line is commonly used to study damage and differentiation in response to redox modifications [54–56]. Previous work has clearly demonstrated the involvement of oxidative stress as one of the main factors in the induction of cell death by palmitate [57–59]; this was confirmed by our experiments where L6 myoblasts were incubated with palmitate for 24 h, resulting in a dramatic decrease in the cell survival and correlating with a significant decrease in the GSH/GSSG ratio.

tBHQ is the metabolite of butylated hydroxyanisole, a synthetic phenolic antioxidant that acts as a redox cycler to generate ROS [60]. Although tBHQ has been widely used as a strong Nrf2 inducer [30, 34, 61], it is known that the expression of genes associated with the antioxidant system is regulated by the Keap1/Nrf2 system. Under basal conditions, Nrf2 is degraded by the ubiquitin-proteasome pathway [37]. However, if the cells are exposed to electrophilic components that modify the redox state, such as tBHQ, Keap1 cysteine residues become oxidized, causing conformational changes that allow Nrf2 dissociation and translocation to the nucleus [60, 62]. Therefore, Nrf2 heterodimerizes with members of the small Maf protein family and binds to the antioxidant/electrophilic response elements (ARE/EpRE). ARE/EpRE promoter encourages the transcription of numerous genes including those involved in glutathione synthesis and recycling (*γ*-GCS, GR), genes with antioxidant properties (HO-1, SOD-1), and genes involved in xenobiotic metabolism and transport [46].

As a result of tBHQ treatment, and as expected, Nrf2 in its active form (pNrf2) was translocated into the nucleus (Figure 5), demonstrating tBHQ ability to activate signaling pathways sensitive to cellular redox changes. Moreover, when L6 myoblasts were treated with tBHQ 50 *μ*M, several changes in redox state were observed. During the first 6 h of treatment, the GSH/GSSG ratio decreased resulting in a more oxidized system; however, from 9 and until 24 h, this ratio increased turning it into a reduced system, therefore giving the cells antioxidant protection to the subsequent palmitate insult.

With regard to the proteins regulated by Nrf2, the results were very interesting because not all of them increased in the same way and at the same time. HO-1 aumented starting at 3 h reaching its highest peak at 6 and 12 h; then, it decreased but still maintained higher levels than the untreated cells, suggesting that it might be participating in the hormetic response. This protein could be related with myoblasts' protection against palmitate because HO-1 is known to activate antioxidant, antiapoptotic, anti-inflammatory, and vasodilatory protective responses [63].

Nrf2 also regulates the transcription of genes related to the GSH system. Here, we found that GST importantly increased at 0.5 and 6 h, while *γ*-GCS and GR augmented only until 12 and 24 h. This could be related to the GST task of eliminating damaged protein at early times after hormesis, whereas *γ*-GCS and GR might be related with GSH synthesis and renewal at later times.

It is very attractive to try to relate the results obtained regarding the activation of Nrf2 mentioned above (Figure 5) and the different changes found in the antioxidant enzyme expression (Figure 8), with the results of the redox state (GSH/GSSG) (Figure 2(c)). Because even though Nrf2 translocates into the nucleus at short times and induces the activation of proteins that will later regulate the redox state, as shown in Figure 8 and as discussed above, not all the enzymes increase their expression at the same time. It is possible to suggest that each of them plays a different role during the protective response; however, their functions could be related in such a way that all of them might be required to induce the full hormetic response. Our data point towards that idea because after a certain time of tBHQ treatment, the redox state (GSH/GSSG) was reestablished (Figure 2(c)), and the protection against the toxic agent became evident (Figure 4(a)). Although our results support this hypothesis, more experiments are required to confirm it.

Ochratoxin has been used in numerous studies as an Nrf2 inhibitor [46, 64]. It inhibits Nrf2 nuclear translocation [65–67] and Nrf2-DNA binding [68]; and it prevents constitutive Nrf2 transcription, due to epigenetic effects [45]. So, in order to confirm Nrf2 participation in the hormetic response activation, OTA was used. Interestingly, our results showed that even though that OTA reduced Nrf2 nuclear translocation (Figure 7(b)), the hormetic response was not completely abrogated (Figure 7(a)), indicating that Nrf2 is not the only transcription factor involved in the tBHQ hormetic effect.

NF-*κ*B is a transcription factor involved in survival and anti-inflammation processes, consisting of five different subunits (RelA, p65, RelB, RelC, p52, and p60), which are able to interact among themselves to form homo- or heterodimers and interact with various promoters, thus transactivating multiple genes [69]. Nevertheless, it has been determined that the subunit p65 is involved in the hormetic response against oxidative stimuli [70]; in this context, we decided to evaluate p65 in L6 myoblasts treated with the tBHQ. Interestingly, we found that NF-*κ*B increases at early times after tBHQ exposure, displaying the same behavior as Nrf2, pointing towards a dual mechanism during the hormetic response. To evaluate NF-*κ*B competence, some proteins regulated by this transcription factor were evaluated. One of them was HSP70 [71], which performs important protection functions and participates in folding and assembly of newly synthesized proteins into macromolecular complexes, aggregation prevention, dissolution and refolding of aggregated proteins, and protein degradation [72]. HSP70 increases at 3 and 6 h after tBHQ treatment; this is an interesting finding because this protein plays a pivotal role in protection against cell death; for instance, blocking HSP70 induces apoptosis [73]. The observed increase in HSP70 could also be related with the protection against protein denaturalization induced by the changes in redox state fomented by tBHQ [74, 75]. So HSP70 might also be participating in cellular protection during the hormetic model.

Another protein regulated by NF-*κ*B which increased after 12 h tBHQ treatment was catalase (CAT), an important antioxidant enzyme that catalyzes the transformation of hydrogen peroxide into water and oxygen. These results support the idea that NF-*κ*B coparticipates with Nrf2 in tBHQ-induced hormetic effect; however, more experiments need to be performed in order to confirm it.

Moreover, since we have previously demonstrated that the hormetic response induced is also regulated by a mechanism that involves the response to DNA damage [76], here we indirectly evaluated that response by determining the expression of the proapoptotic protein Bax. It is known that Bax is regulated by the DNA damage sensor p53 [77]. Our results showed that Bax increased at 0.5 and 1 h after tBHQ treatment, and subsequently, the expression gradually decreases after 3 h. This decrease is consistent with Nrf2 and NF-*κ*B nuclear translocation timing, suggesting that hormetic treatment activates other molecular processes involved in DNA protection and repair in which p53 and other proteins of the Bcl-2 family might be involved.

As mentioned before classical hormesis is as follows: “a process in which exposure to a low dose of a chemical agent or environmental factor that is damaging at higher doses induces an adaptive beneficial effect on the cell or organism” [25, 26]. In other words, it could be said that hormesis is an adaptive mechanism that a cell or organism has developed to compensate for any imbalance in its homeostasis, caused by the exposure to factors that generate stress. In this way, when we pretreated L6 myoblasts with tBHQ, a hormetic response was induced by activating molecular pathways that gave protection to the cells for a subsequent stressful exposure (palmitate). This is very interesting because it opens the door to study interventions with molecules that are not classic antioxidants but that induce a hormetic effect and that could have therapeutic purposes in preventing muscle damage and reducing oxidative damage induced by a high-fat diet.

In summary, our study provides evidence that tBHQ can confer protection in L6 myoblasts against the toxicity induced by palmitate due to a dual activation of the Nrf2 and NF-*κ*B signaling pathways.

## Data Availability

No data were used to support this study, beside the ones presented in this paper.

## Supplementary Materials

Supplementary Materials A. Cellsurvival was determined after OTA treatment for 24 h (2, 5, 10, 20, 50, and 100 *μ*M). Each bar represents the mean ± S.E. of 9 determinations performed in three independent experiments. Statistical significance with respect to untreated cells ^∗^*p* < 0.05. B. Representative immunofluorescence images after OTA treatment for 24 h (2, 5, and 10 *μ*M). pNrf2 (red) and DAPI (blue).
