# Supplementary figures and images for: tBHQ Induces a Hormetic Response That Protects L6 Myoblasts against the Toxic Effect of Palmitate
Source: Oxid Med Cell Longev. 2020 May 16;2020:3123268. doi: 10.1155/2020/3123268 (PMC7246405; doi:10.1155/2020/3123268)

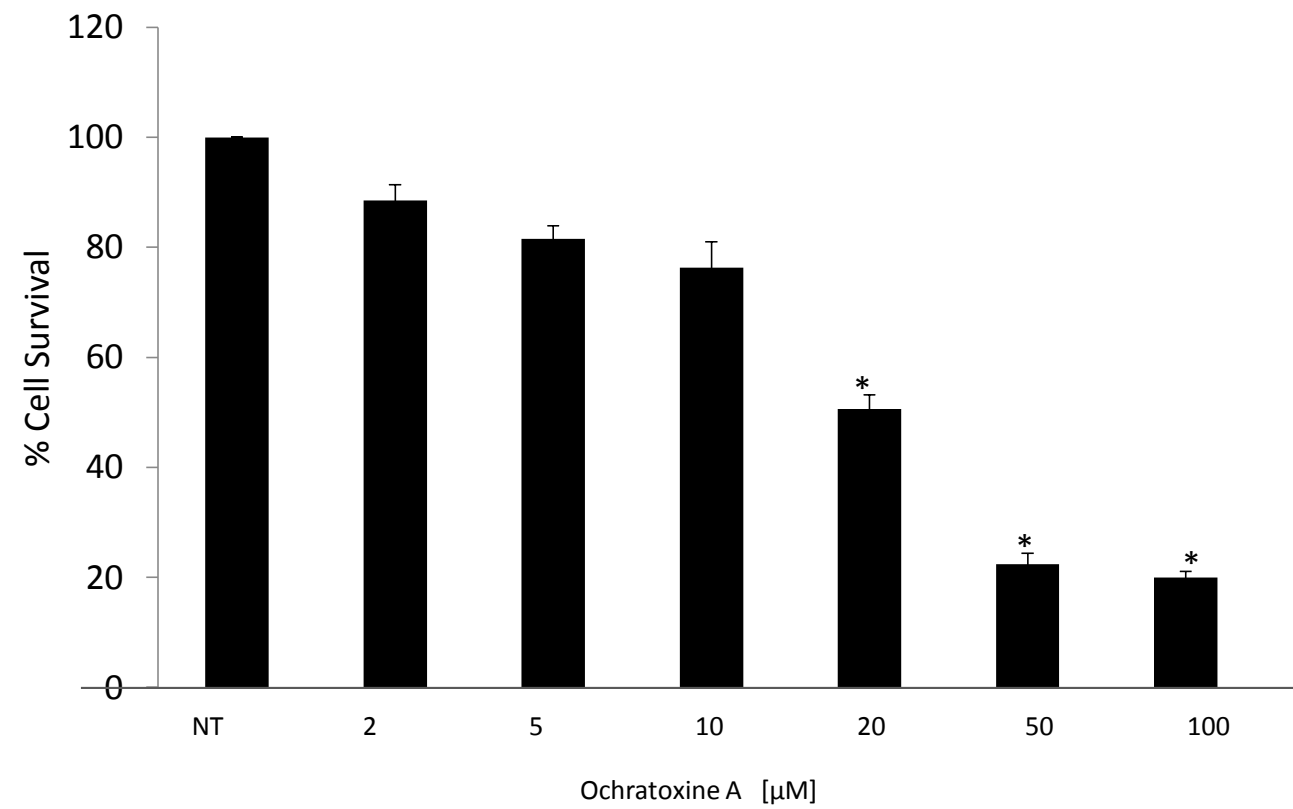

Supplementary FIGURE 1 A

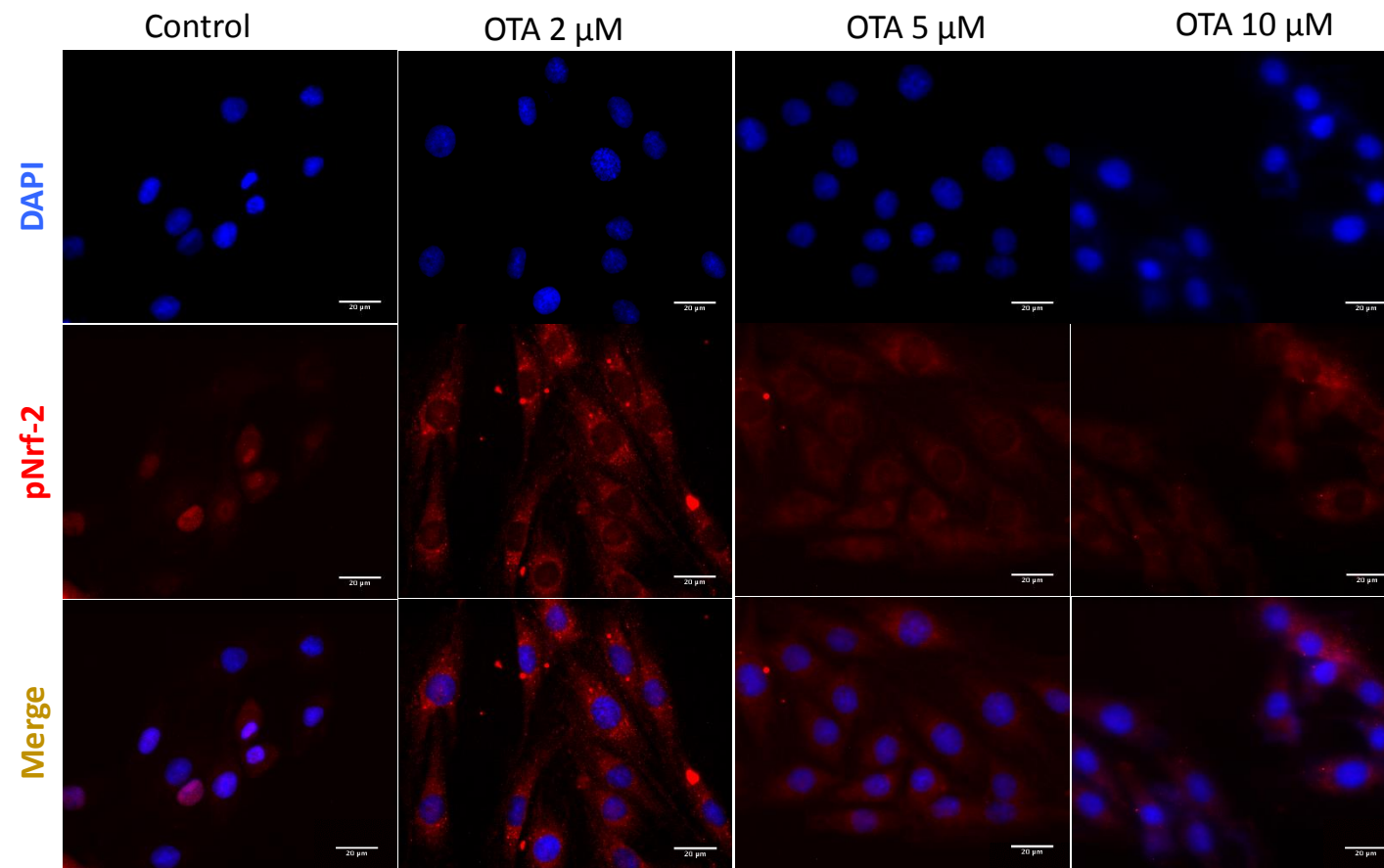

Supplementary FIGURE 1 B

Supplement: Supplementary Materials — A. Cellsurvival was determined after OTA treatment for 24 h (2, 5, 10, 20, 50, and 100 μM). Each bar represents the mean ± S.E. of 9 determinations performed in three independent experiments. Statistical significance with respect to untreated cells ∗p < 0.05. B. Representative immunofluorescence images after OTA treatment for 24 h (2, 5, and 10 μM). pNrf2 (red) and DAPI (blue). [file 3123268.f1.pdf]
